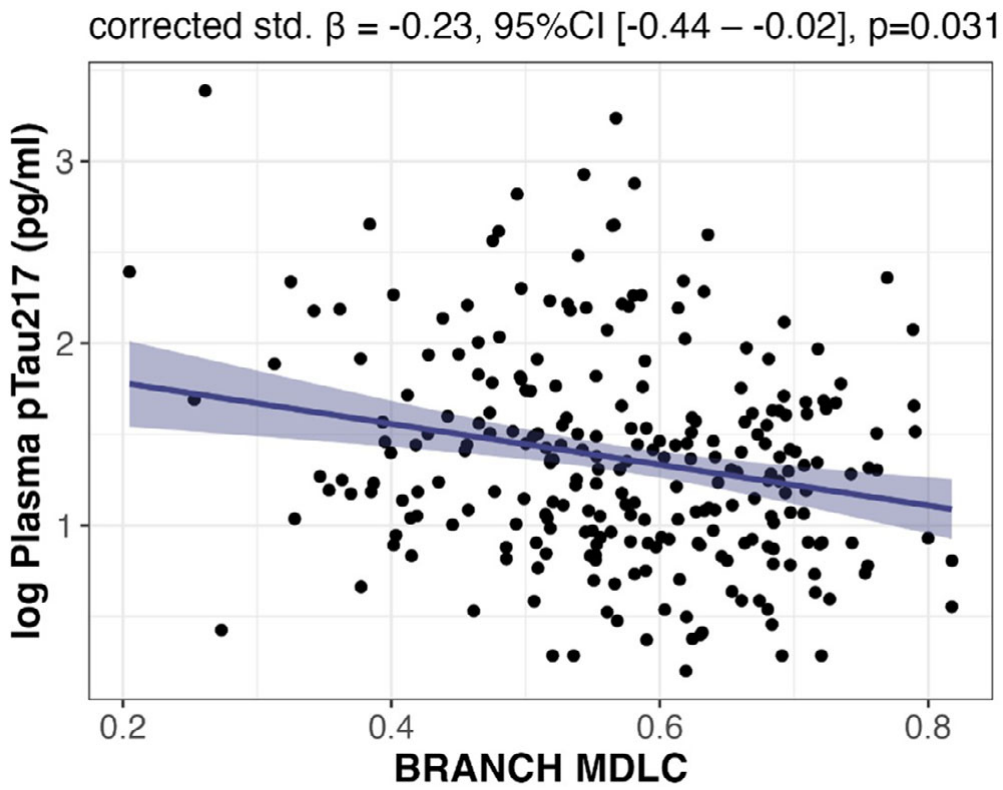# Remote digital multi‐day learning curves detect subtle cognitive differences associated with plasma *p*‐tau217 among cognitively unimpaired older adults

**DOI:** 10.1002/alz70857_106782

**Published:** 2025-12-24

**Authors:** Roos J Jutten, Daniel Soberanes, Hairin Kim, Mark A. Dubbelman, Lia D'Aquila, Hyun‐Sik Yang, Deborah Blacker, Dorene M. Rentz, Gad A. Marshall, Keith A. Johnson, Reisa A. Sperling, Rebecca E. Amariglio, Kathryn V Papp

**Affiliations:** ^1^ Massachusetts General Hospital, Harvard Medical School, Boston, MA, USA; ^2^ Brigham and Women's Hospital, Harvard Medical School, Boston, MA, USA; ^3^ Department of Epidemiology, Harvard T. H. Chan School of Public Health, Boston, MA, USA

## Abstract

**Background:**

Timely identification of individuals at‐risk for Alzheimer's disease (AD) is pivotal for secondary prevention and requires innovative approaches. Combining remote smartphone‐based cognitive assessments with blood‐based biomarkers holds promise for both sensitive and scalable detection of early AD‐related cognitive changes. Here, we aimed to investigate whether the Boston Remote Cognitive Assessment of NeuroCognitive Health (BRANCH) captures cognitive changes associated with early AD pathophysiology as measured by plasma *p*‐tau217.

**Method:**

*N* = 254 cognitively unimpaired older adults (age=74.5±8.6, 68% female, 16.6±2.4 years of education) from four well‐characterized cohorts completed multi‐day BRANCH on their personal device. Multiday BRANCH includes two associative memory tests (Face Name and Groceries Prices) and a processing speed test with an associative memory component (Digit Signs) with identical stimuli repeated for seven consecutive days. For each test, an MDLC score was computed using an area under the curve method combining day 1 performance with a non‐linear learning trajectory over the subsequent six days. MDLCs for each individual test were averaged into a BRANCH Composite MDLC. All cohorts had standardized in‐clinic cognitive test data available, from which a Preclinical Alzheimer's Cognitive Composite (PACC‐5) score was derived. Concentrations of plasma *p*‐tau217 were measured using the Meso Scale Discovery platform. Linear regression models adjusting for age, sex, years of education and study cohort were used to investigate the association between *p*‐tau217 (log‐transformed values) and BRANCH MDLC scores. For comparison, similar analyses were run with PACC‐5 scores and *p*‐tau217.

**Result:**

Lower BRANCH Composite MDLC scores were associated with higher *p*‐tau217 levels (corrected std. β = ‐0.23, 95%CI [‐0.44 – ‐0.02], *p* = 0.031) (Figure 1), which was primarily driven by the Digit Signs test (corrected std. β = ‐0.22, 95%CI [‐0.42 – ‐0.02], *p* = 0.031). In contrast, we did not find an association between the PACC‐5 and *p*‐tau217 (corrected std. β = ‐0.15, 95%CI [‐0.37 – 0.07], *p* = 0.187).

**Conclusion:**

These results complement our previous work that multi‐day BRANCH may improve the detection of very subtle memory deficits that are associated with early AD pathophysiology. Combining a remote and sensitive cognitive paradigm like BRANCH with plasma biomarkers may facilitate scalable detection of those at risk for AD‐related cognitive decline.